# Data on alpine grassland diversity in Gran Paradiso National Park, Italy

**DOI:** 10.1016/j.dib.2019.103942

**Published:** 2019-04-23

**Authors:** Samuel Hoffmann, Laura Steiner, Andreas H. Schweiger, Alessandro Chiarucci, Jonas Benner, Anotnello Provenzale, Carl Beierkuhnlein

**Affiliations:** a University of Bayreuth, Biogeography, Universitätsstr. 30, D-95447, Bayreuth, Germany; b Büro für Ökologische Studien, BfÖS, Oberkonnersreuther Str. 6a, D-95448, Bayreuth, Germany; c University of Bayreuth, Plant Ecology, Universitätsstr. 30, D-95447, Bayreuth, Germany; d Bayreuth Center of Ecology and Environmental Research, BayCEER, Universitätsstr. 30, D-95447, Bayreuth, Germany; e Department of Biological, Geological, and Environmental Sciences, Alma Mater Studiorum – University of Bologna, Via Irnerio 42, 40126, Bologna, Italy; f Institute of Geosciences and Earth Resources, National Research Council of Italy, Via Moruzzi 1, 56124, Pisa, Italy; g Geographical Institute Bayreuth, GIB, Universitätsstr. 30, D-95447, Bayreuth, Germany

**Keywords:** Alpine grassland, Species diversity, Plot data, Cover, Abundance, Vegetation survey, Vegetation monitoring

## Abstract

The diversity of alpine grassland species and their functional traits constitute alpine ecosystem functioning and services that support human-wellbeing. However, alpine grassland diversity is threatened by land use and climate change. Field surveys and monitoring are necessary to understand and preserve such endangered ecosystems. Here we describe data on abundances (percentage cover) of 247 alpine plant species (including mosses and lichens) inside nine 20 m by 20 m plots that were subdivided into 2 m by 2 m subplots. The nine plots are located in Gran Paradiso National Park, Italy. They cover three distinct alpine vegetation subtypes (‘pure’ natural grassland, sparsely vegetated ‘rocky’ grassland, and wetland) in each of three valleys (Bardoney, Colle de Nivolet and Levionaz) between 2200 and 2700 m a.s.l., i.e. above the treeline. The vegetation survey was conducted in 2015 at the peak of vegetation development during August. The dataset is provided as supplementary material and associated with the research article “Optimizing sampling effort and information content of biodiversity surveys: a case study of alpine grassland” [1]. See [1] for data interpretation.

**Table:** Specifications Table

Subject area	*Biogeography, vegetation ecology, biodiversity conservation*
More specific subject area	*Community ecology*
Type of data	*Plant species cover-abundance within a quadratic and gridded plot design*
How data was acquired	*Field observation*
Data format	*Table*
Experimental factors	*Mosses and lichens are included. The cover of litter, deadwood, bare soil, rocks, gravel and water are given. Uncertain species identities are noted.*
Experimental features	*Two independent observers estimated the cover-abundance of each plant species. The mean of these two estimates is given.*
Data source location	*Gran Paradiso National Park, Italian Alps*
Data accessibility	*The dataset is provided as*supplementary material*.*
Related research article	*S. Hoffmann, L. Steiner, A.H. Schweiger, A. Chiarucci, C. Beierkuhnlein, Optimizing sampling effort and information content of biodiversity surveys: a case study of alpine grassland, Ecol. Inform. 51 (2019) 112–120.*https://doi.org/10.1016/J.ECOINF.2019.03.003. [1]

**Table:** 

**Value of the Data** • Since alpine plant diversity is threatened by land use and climate change but provides ecosystem functioning and services [2], [3], vegetation surveys and monitoring of the endangered vegetation types above the treeline are needed to inform conservation management and policy. • The sampling design and data allows for various investigations on the relationship between sampling scale and species diversity (e.g. Refs. [4], [5]). • Due to the sampling design the dataset is suitable to be linked to remote sensing data in order to analyze the relationship between plant species diversity and spectral signals (e.g. Ref. [6]). Such investigations can facilitate large scale vegetation mapping by relatively low effort [7]. • The data can be integrated into macroecological analyses.

## 1 Data

**Fig. 1:**
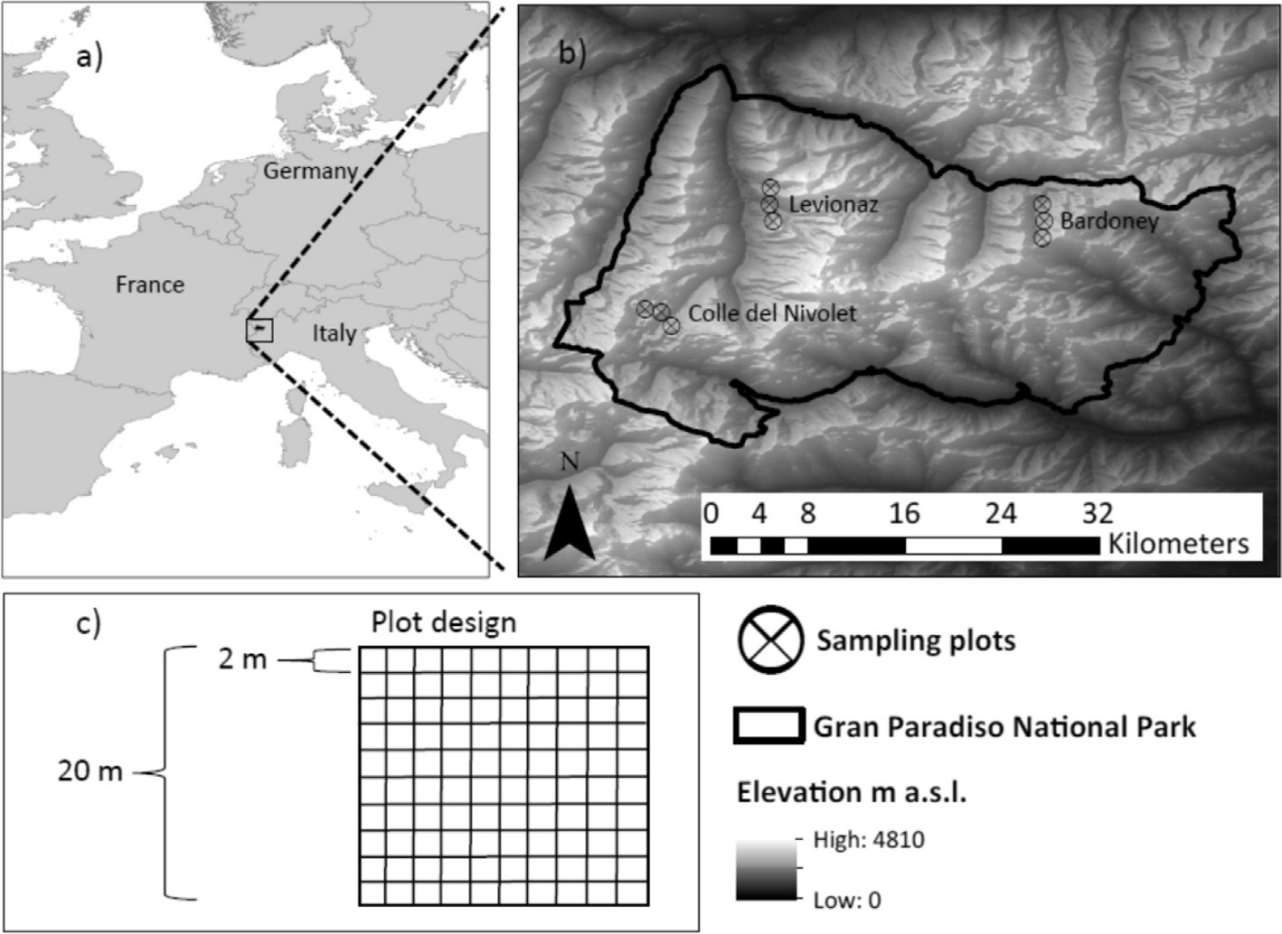
Geographical location of the study area. a) Gran Paradiso National Park is located in the European Alps, northwestern Italy. b) Nine sampling plots were established, three in each of the three alpine grassland subtypes inside each of three valleys (Colle del Nivolet, Levionaz, and Bardoney). c) The sampling plot was designed as a 20 m by 20 m quadratic square subdivided into 100 subplots of 2 m by 2 m. Figure adapted from Ref. [1].

The data presented here involves cover-abundance of alpine grassland species in Gran Paradiso National Park, Italy (Fig. 1a and b). We estimated percentage cover of plant species (including mosses and lichens), litter, deadwood, bare soil, rocks, gravel and water inside nine 20 m by 20 m plots that were subdivided into 100 2 m by 2 m subplots (Fig. 1c). The nine plots cover three vegetation subtypes (‘pure’ natural grassland, sparsely vegetated ‘rocky’ grassland, and wetland) in each of three valleys (Bardoney, Colle de Nivolet, and Levionaz) between 2200 and 2700 m a.s.l. (Fig. 1b). The vegetation survey was conducted in the middle of the yearly vegetation period during August 2015. The plots' boundary lines were North-South and East-West aligned according to Universal Transverse Mercator reference system (UTM Zone 32N) coordinates. The Global Positioning System (GPS) coordinates of the plots' corner points are given in the UTM zone 32N. The dataset is given as supplementary material and is additionally registered at the Dynamic Ecological Information Management System - Site and Dataset Registry (DEIMS-SDR) [8] under the UUID b549ff14-f40f-4749-8e2f-f16f6e523753 (see https://deims.org/dataset/b549ff14-f40f-4749-8e2f-f16f6e523753).

## 2 Experimental design, materials, and methods

We established nine quadratic plots in the Gran Paradiso National Park in northwestern Italy (Fig. 1a). Quadrats mitigate the confounding effect of environmental heterogeneity on species diversity [9]. The plots cover three subtypes of alpine vegetation that were located with the support of the CORINE Land Cover map from 2012 (available at https://land.copernicus.eu/pan-european/corine-land-cover) and expert knowledge. The three vegetation subtypes are ‘pure’ natural grassland, sparsely vegetated ‘rocky’ grassland, and ‘wet’ grassland (wetlands). Each vegetation subtype was sampled in each of three valleys (Bardoney, Colle de Nivolet, and Levionaz; Fig. 1b), which resulted in one plot per vegetation subtype and valley. The plots were established on flat terrain. Each of the nine plots had an extent of 20 m by 20 m (400 m^2^) and was subdivided into 100 subplots measuring 2 m by 2 m (Fig. 1c).

During the fieldwork, the plot boundaries were marked with cord and the plot corners with four pegs. The first two pegs were aligned to North-South with a compass. The aberration between 32 UTM to North in the Aosta valley is −2.33°. The aberration between North and the magnetic North is −2°. To compensate these aberrations, we revised the North on the compass with −4°. The quadratic squares were built by three measuring tapes and trigonometric functions.

The percentage cover-abundance of each plant species (including mosses and lichens), litter, dead wood, bare soil, rock, gravel and water were estimated for each subplot. Ordinal cover estimates from 0 to 100% were done independently by two people to reduce observer bias [10]. We then took the mean of these two cover estimates that was rounded to no decimal places. Plants with a cover of less than one percent were set to 0.5% cover for simplification of statistical analyses. The vegetation survey was conducted in the middle of the yearly vegetation period during August 2015. Species were identified using ‘Flora Helvetica’ [11], ‘Flora Vegetativa’ [12], ‘Flora Alpina’ [13] and ‘Guida alla flora della Valle d’Aosta’ [14]. The taxonomic names of species were standardized using TCL-function in R package “taxonstand” [15], which refers to “The Plant List” website (www.theplantlist.org). The resulting taxonomic information are provided as a separate table in addition to the plot data.

Moreover, the plots were established on flat terrain and the plot boundaries were North-South and East-West aligned based on UTM-coordinates in reference system WGS 84, i.e. the subplots IDs are ordered from the North-West corner to the South-East corner. GPS coordinates were taken at each plot corner. We used Garmin Montana 600 to determine the GPS coordinates of the plots' corner points. To get the date and time of the strongest GPS signal (i.e., most satellites available at position), the GPS-satellites’ Almanach data was queried. Waypoint averaging was applied to improve the positioning accuracy of the Garmin Montana 600. The position accuracy is given in meters. Additionally, all corners were marked by burying magnets in a soil depth of about 20 cm. This allows precise relocation and long-term monitoring.

## Funding

This work was supported by the European H2020 Project ECOPOTENTIAL, grant agreement No. 641762. The authors have no competing interests.
